# Application of high-precision 3D scanner in keloids evaluation to improve patients’ compliance: a questionnaire-based study

**DOI:** 10.1186/s12967-024-05079-w

**Published:** 2024-04-15

**Authors:** Huayi Wu, Zixi Jiang, Xiang Chen, Shuang Zhao, Zeyu Chen

**Affiliations:** 1 School of Mechanical and Electrical Engineering, Central South University, Changsha, 410083 China; 2 Department of Dermatology, Xiangya Hospital, Central South University, 87 Xiangya Road, Kaifu District, Changsha, Hunan China; 3 National Clinical Research Center for Geriatric Disorders, Xiangya Hospital, Central South University, Changsha, 410008 Hunan China

**To the Editor,**

Keloid is a prevalent benign skin neoplasm. Each year in the developed world, approximately 100 million people suffer from scar-related issues [1]. Current guidelines recommend that the combination of 5-fluorouracil (5-FU) and triamcinolone acetonide (TA) injections, administered in 3–5 treatment sessions (6–10 weeks), effectively reduces the elevation and redness of keloids [2, 3]. Consistently, the effectiveness of treating keloids, whatever evaluated by naked-eye observations or Vancouver Scar Scale (VSS), is observed after at least three treatment sessions in previous observational studies [4]. However, as patients are unable to obtain positive feedback on the first two treatment sessions of keloid treatment (within 4 weeks) through naked-eye observations or VSS scores, and the treatment process is intensely painful, leading to poor patient compliance and suspension of treatment. Therefore, a new approach that can precisely reflect the unconspicuous efficacy during the previous two treatment sessions and encourage patients to adhere to the therapy is crucial. In this study, we aim to investigate whether the application of a high-precision 3D scanner in measuring the volume change of keloids after treatment can improve patient compliance towards intralesional 5-FU combined with TA.

As shown in Additional file 1: Figure S1, the data collection has been carried out with the consent of the patients and has obtained approval from the Medical Ethics Committee of Xiangya Hospital, Central South University. As shown in Additional file 1: Figure S2, we conducted accuracy testing on the 3D scanner, and the results indicated that the scanner exhibited high precision in imaging keloids-like objects. The schematic diagram of the scanning process, data reconstruction and volume calculation was showed in Fig. 1. A high-precision 3D scanner (iReal 2E, SCANTECH, China) was applied to collect the 3D image data of keloids and the 3D image data was reconstructed by using iReal 3D software. We performed image segmentation and volume calculation on scar data based on three-dimensional point cloud technology. (CloudCompare, TELECOM ParisTech). Keloids data from nine patiens was collected using a high-precision 3D scanner. The matched traditional clinical images, 3D clinical images, VSS score and relative alterations of keloid volume of nine cases (1 session: 3 cases; 2 sessions: 3 cases; 3 sessions: 3 cases) pre- and post-treatment were presented in a web-based questionnaire. Forty-five keloid patients were questioned whether they are willing to undergo this therapy which was blinded to them based on different efficacy presenting model.

**Fig. 1:**
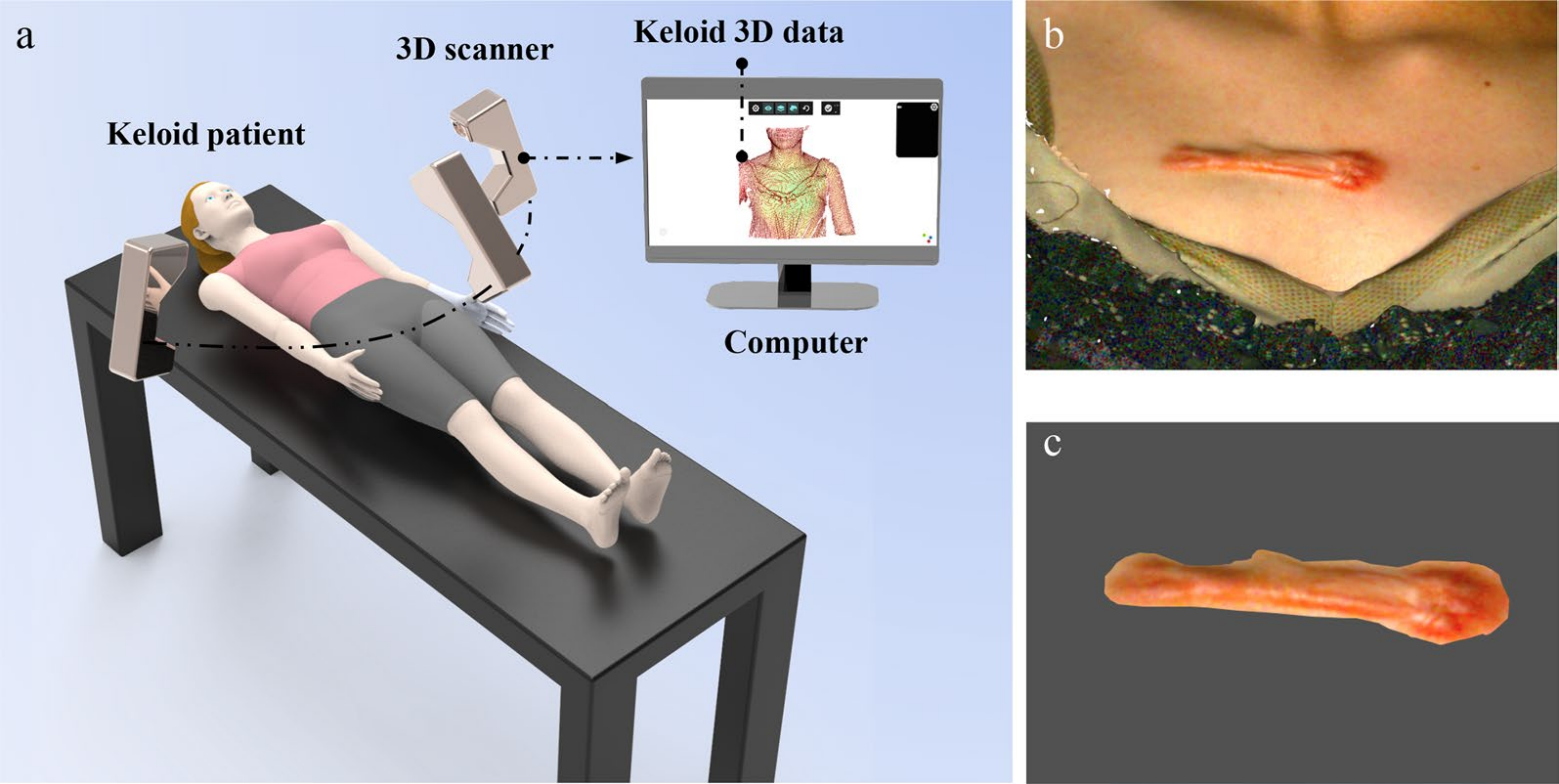
**a** Schematic diagram of the scanning process. **b** 3D keloid image reconstruction. **c** 3D keloid image segmentation and volume calculation

The results were suggested in Fig. 2. Compared with traditional clinical images, 3D clinical images and VSS score, the average willingness rate (AWR) of relative volume data achieved the highest percentage, which to one, two and three treatment sessions was 44.4%, 54.8% and 48.1%, respectively. As the VSS score started to decrease since the third treatment session, the AWR towards 3 treatments (42.4%) was higher than both 1 session (38.5%) and 2 sessions (39.3%). Detailed information concerning the questionnaire was presented in Additional file 1, and the detailed information concerning the questionnaire response sheet was presented in Additional file 2.

**Fig. 2:**
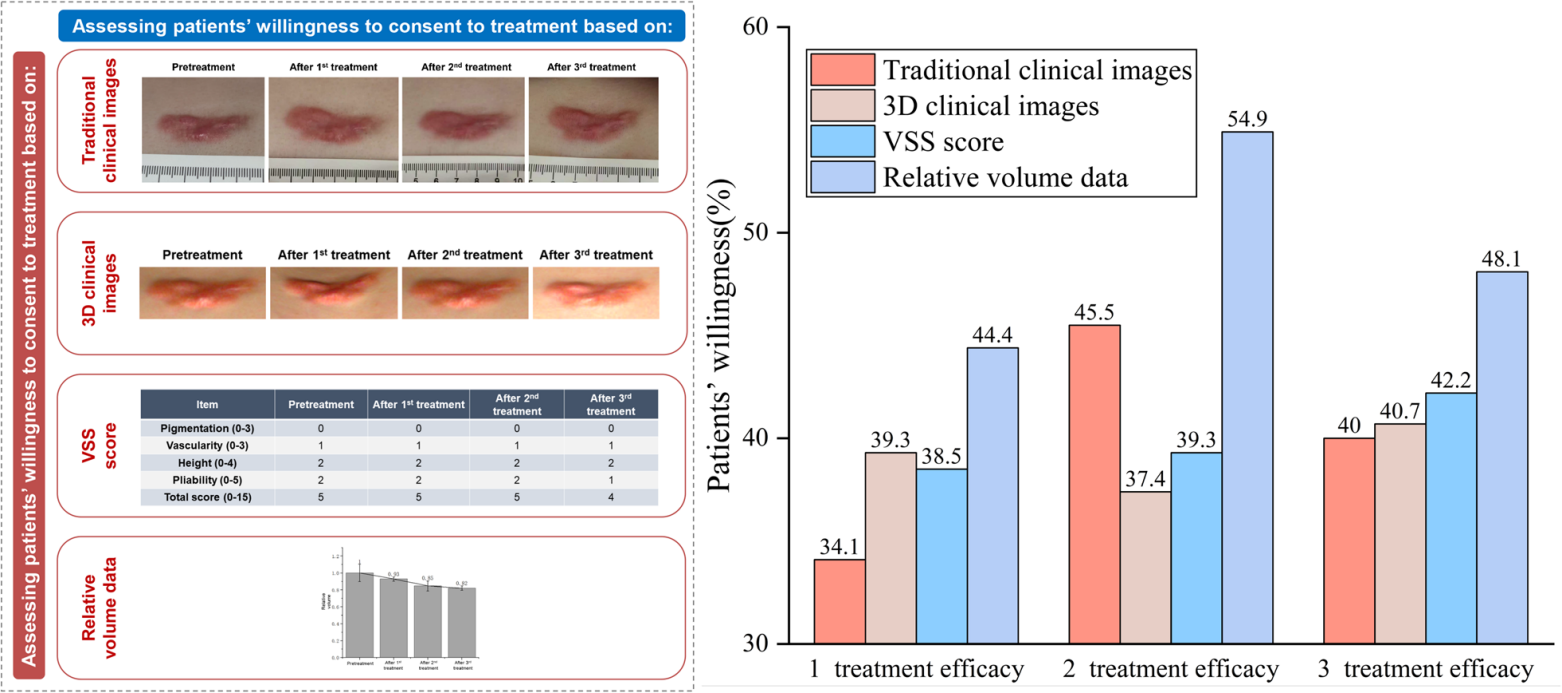
Assessing patients’ willingness to treatment based on different types of images/data and number of treatments

Collectively, these results show that making patients aware of the relative volume change of keloids via the application of a high-precision 3D scanner can significantly improve patients’ compliance with treatment. Utilizing a high-precision 3D scanner can more accurately assist in evaluating the effectiveness of keloid treatment, but it requires further confirmation through large-scale, multicenter clinical studies. Notably, none of the AWR is higher than 60% which indicates keloid patients are still not completely satisfied with the efficacy of 3 sessions of intralesional 5-FU combined with TA. It is necessary for us dermatologists to make long-term physical and psychological treatment schedules for keloid patients (Additional file 2).

### Supplementary Information

**Additional file 1:** Supplementary Material 1.

**Additional file 2:** Supplementary Material 2.

## Data Availability

The datasets used and/or analyzed during the current study are available from the corresponding author on reasonable request.
