# Supplementary material for: Application of high-precision 3D scanner in keloids evaluation to improve patients’ compliance: a questionnaire-based study
Source: J Transl Med. 2024 Apr 15;22:354. doi: 10.1186/s12967-024-05079-w (PMC11020470; doi:10.1186/s12967-024-05079-w)
Supplement: Supplementary file 1 — Additional file 1: Supplementary Material 1. [file 12967_2024_5079_MOESM1_ESM.pdf]

Supplementary Material 1

Title: Application of High-Precision 3D Scanner in Keloids Evaluation to Improve Patients' Compliance: A Questionnaire-Based Study.

Huayi Wu[1, #]Ph.D, Zixi Jiang[2,3, #]M.D., Ph.D, Xiang Chen[2,3] M.D., Ph. D, Shuang Zhao[2,3]M.D., Ph. D, Zeyu Chen[1,2,3]Ph.D.

[1]School of Mechanical and Electrical Engineering, Central South University, Changsha 410083,
China
[2]Department of Dermatology, Hunan Engineering Research Center of Skin Health and Disease, Hunan Key Laboratory of Skin Cancer and Psoriasis, Xiangya Hospital, Central South University, Changsha, Hunan, China.
[3]National Clinical Research Center for Geriatric Disorders, Xiangya Hospital, Central South University, Changsha, Hunan 410008, China.
Huayi Wu and Zixi Jiang are co-first authors.

Corresponding author:
Zeyu Chen Ph.D.
School of Mechanical and Electrical Engineering, Central South University, Changsha 410083,China.
Email address: zeyuChe@csu.edu.cn
Shuang Zhao M.D., Ph.D.
Department of Dermatology, Xiangya Hospital of Central South University, 87 Xiangya Road, Kaifu District, Changsha, Hunan Province, China.
E-mail: shuangxy@csu.edu.cn

Contents

1. **Ethical materials**
2. **Accuracy assessment of three-dimensional scanner**
3. **Questionnaire**

1. Ethical materials:

1

中南大学湘雅医院医学伦理委员会

Medical Ethics Committee of Xiangya Hospital Central South University

伦理审查意见函

Ethic Review Comment Letter

审查日期 Review date：2023 年 08 月 01 日　　　　伦审科快第（202308636） 号

项目名称 Project name	皮肤肿瘤、银屑病和变态反应性皮肤病患者生物样本和临床信息采集		
项目类别 Classification	科研项目	项目来源 Project source	研究者发起
审查方式 Review form	快速审查	审查类别 Review category	复审
负责科室 Responsible department	中南大学湘雅医院皮肤科	我院研究项目负责人 Principal investigator	粟娟/主任医师
审查文件及版本号 File and Version number	详见附件一		
快速审查 Quick review	委员： 2 人 ：姓名：张江林、刘星		

投票结果 Voting result	批准（2） Consent	修改后批准（0） Modified consent	不批准（0） Disagree	暂停或终止已批准的研究（0） Pause or termination

评审意见 Review comment	审查决定（Review conclusion）：批准。	
	主任委员/副主任委员（Chairman/Vice-Chairman）：[illegible]	签发日期（Issuing date）：2023 年 8月 04日
年度/定期跟踪审查频率 Annual /regular tracking frequency	12 个月	
伦理委员会 Ethics Committee	中南大学湘雅医院临床医学伦理委员会	
有效期：Expiry date	2024 年 08 月04日	

备注：本伦理委员会的职责、人员构成、运行和记录遵循《药物临床试验伦理审查工作指导原则》（2010）、《涉及人的生物医学研究伦理审查办法》（2016）、《医疗器械临床试验质量管理规范》（2016）、GCP（2020）、ICH-GCP、等相关法规。

The medical ethics committee's responsibilities, composition, operations and records are fully compliant with "Guidelines for ethical review of drug clinical trials"(2010)、"Methods for ethical review of biomedical research involving human beings"（2016）、"Standard for quality control of clinical trials of medical devices"（2016）、GCP（2020）、ICH-GCP and other related regulations.

地址：湖南省长沙市湘雅路 87 号　　邮编：410008　　电话号码：86-731-84327919
Address：No.87 Xiangya Road ,Changsha(410008) Hunan　　Tel：86- 731-84327919
E-mail:xyyyllwyh @126.com

Figure S1. Ethic review comment letter

2. Accuracy assessment of three-dimensional scanner

Methods: In this experiment, two small objects were created using modeling software to simulate patient scars, each with a distinct shape. The volumetric accuracy of the 3D scanner was evaluated by scanning these objects. The design model was manufactured using a high-precision 3D printer. The actual size of the manufactured model was measured using vernier calipers, and its volume was calculated. The high-precision 3D scanner was used to scan the manufactured objects. The 3D scanning data was reconstructed and utilized to calculate the volume. The error is calculated by comparing the difference between the volume of the 3D scan data and the actual measured volume; dividing the difference by the actual measured volume. By subtracting the error from 1, we measured the volumetric accuracy of the 3D scanner in this experiment. Each model was measured and scanned three times in parallel.

Results: The design model is as follows: Semicircle: radius of 5 mm; Rectangle: 10 mm×10 mm× 5mm; The average volume of the measured semicircle model is 263.61 mm^3, with a standard deviation of 2.11; The average volume of the measured rectangle model is 494.39 mm^3, with a standard deviation of 2.65. The volume of the semicircular model measured by the 3D scanner is 273.09 mm^3, with a standard deviation of 18; the volume of the rectangular model is 474.28 mm^3, with a standard deviation of 8.52. The calculation results show that the accuracy of the rectangular model measured by the 3D scanner is 96.4%, the accuracy of the rectangular model measured by the 3D scanner is 95.9%, and the average accuracy is 96.2%.

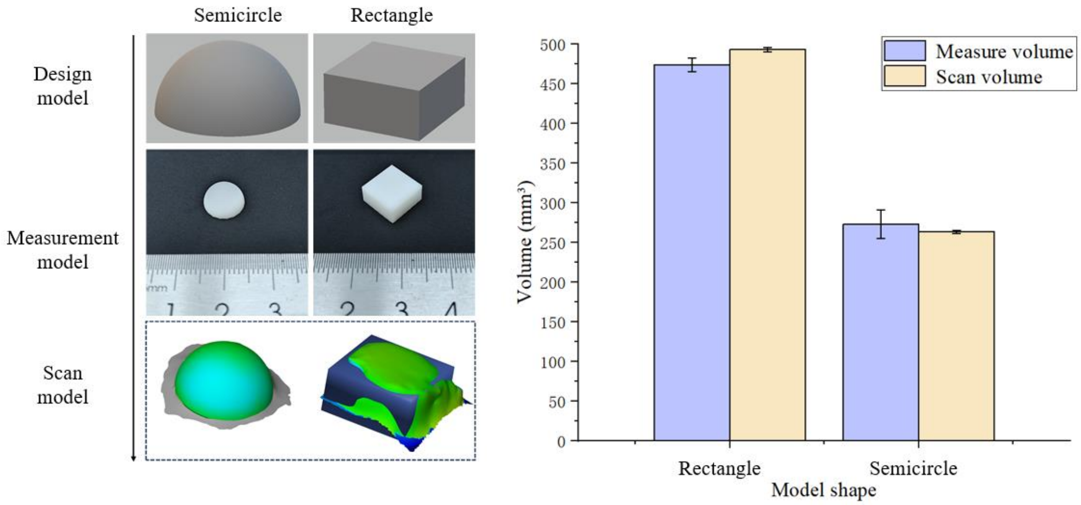

Figure S2. Results of high-precision 3D scanner accuracy test

3. Questionnaire:

Medical compliance assessment of Keloids treatment

Keloids are benign skin tumors characterized by treatment resistance and a high recurrence rate. Current recommendations involve using a combination of methods for scar treatment. However, patients often exhibit low medical compliance due to the difficulty in observing treatment efficacy, leading to treatment discontinuation. This questionnaire aims to explore factors influencing the medical compliance of scar patients. The process of filling out the questionnaire will take approximately 3 minutes of your time. Thank you very much for your participation!

PART 1 Basic patient information

1. Gender [multiple choice] *

○ male

○ female

2. Age [multiple choice] *

○ under 20 years

○ 20-29 years

○ 30-39 years

○ 40-49 years

○ 50-59 years

○ Over 60 years

3. Education background [multiple choice] *

○ Primary school

○ Junior high schoo

○ Senior high school

○ Technical secondary school

○ Undergraduate

○ Junior colleges

○ Graduate

○ Ph.D

○ Other __________________

4. Place of family residence [multiple choice] *

○ Town

○ Contryside

5. Are you a patient with hypertrophic scar/keloid？ [multiple choice] *

○ No

○ Hypertrophic scar

○ Keloid

6. Where are your hypertrophic scars or keloids located on your body？ [multiple response] *

□ Head and face

□ Neck

□ Chest

□ Abdomen

□ Back

□ Perineum

□ Buttocks

□ Arm

□ Hand

□ Lower limbs

□ Feet

□ Other __________________

Depending on the 2nd or 3rd option from question 5.

7. How long have you been suffering from hypertrophic scars/keloids？ [Fill in the blanks] *
(Please fill in Arabic numerals, for example: if you have had keloids for 3 years, write "3"; if you have had hypertrophic scars for 6 months, write "0.5".）

____

Depending on the options provided in question 6, which are 1, 2, 3, 4, 5, 6, 7, 8, 9, 10, 11, or 12.

PART 2 Quality control

8. Among the following pictures, one of them is a scar tissue, and one of them is normal skin. Please select the picture of normal skin. [multiple choice] *

○ Picture 1

○ Picture 2

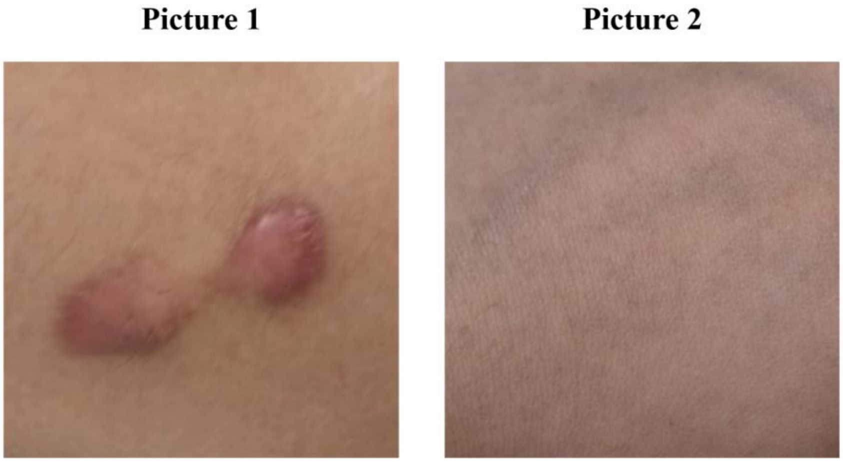

PART 3 Medical assessment

9. If you are a scar patient, there is a treatment method that results in the effect shown in the picture after treatment, would you be willing to continue the treatment?

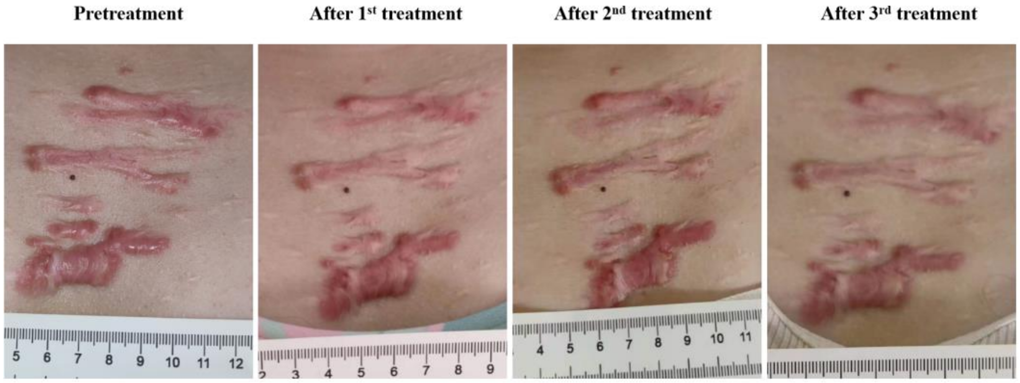

[Enter a number from 0 (very unwilling) to 10 (very willing)]*

____

10. If you are a scar patient, there is a treatment method that results in the effect shown in the picture after treatment, would you be willing to continue the treatment ?

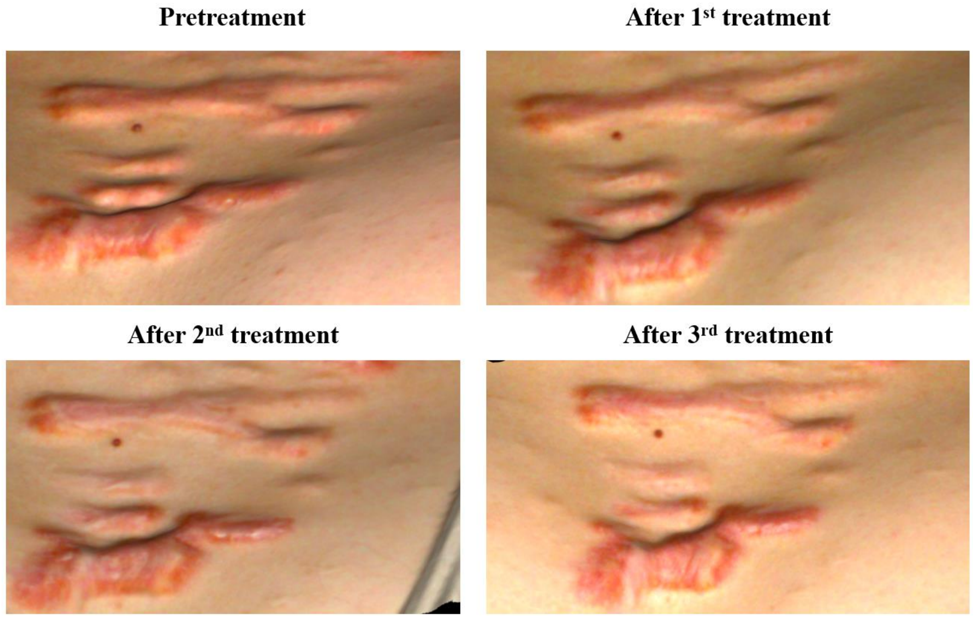

[Enter a number from 0 (very unwilling) to 10 (very willing)]*

____

11. If you are a scar patient, there is a treatment method that results in the effect shown in the picture after treatment, would you be willing to continue the treatment ?

VSS score

(Note: Note: VSS score is a commonly used clinical rating for the severity of scars. The higher the total score, the more severe the scar)

VSS Score Items	Pretreatment	After 1st treatment	After 2nd treatment	After 3rd treatment
**Pigmentation (0- Close to skin color, 3- Darker color)**	1	1	1	1
**Vascularity (0- Close to skin color, 3- Bluish)**	2	2	2	2
**Height (0- Normal, 4- >4mm)**	3	3	3	2
**Pliability (0- Normal, 5- Contraction causing functional impairment)**	2	2	1	1
**Total Score**	8	8	7	6

[Enter a number from 0 (very unwilling) to 10 (very willing)]*

____

12. If you are a scar patient, there is a treatment method that results in the effect shown in the picture after treatment, would you be willing to continue the treatment ?

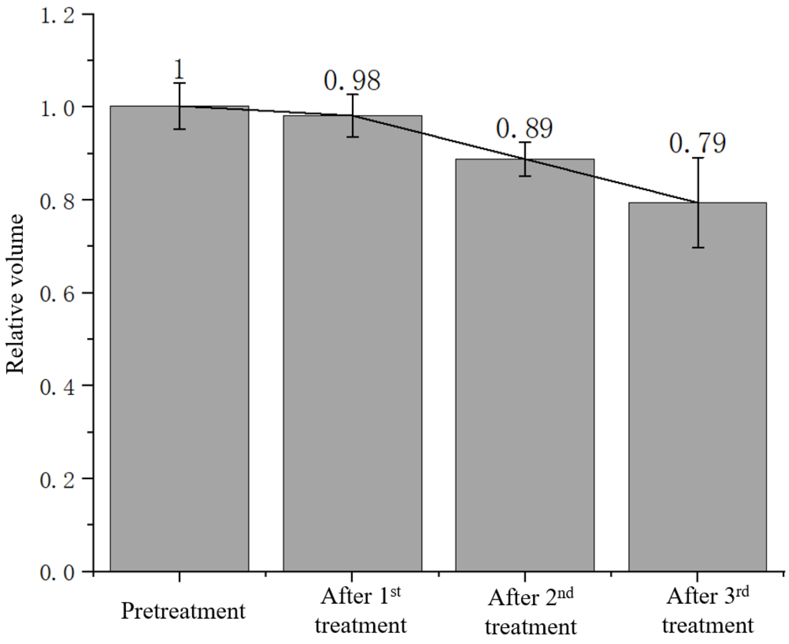

[Enter a number from 0 (very unwilling) to 10 (very willing)]*

____

13. If you are a scar patient, there is a treatment method that results in the effect shown in the picture after treatment, would you be willing to continue the treatment ?

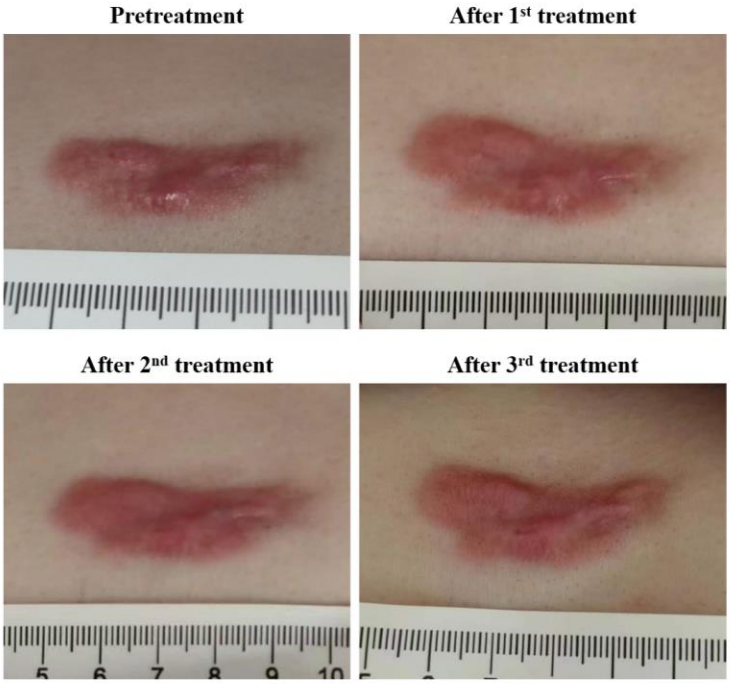

[Enter a number from 0 (very unwilling) to 10 (very willing)]*

____

14. If you are a scar patient, there is a treatment method that results in the effect shown in the picture after treatment, would you be willing to continue the treatment ?

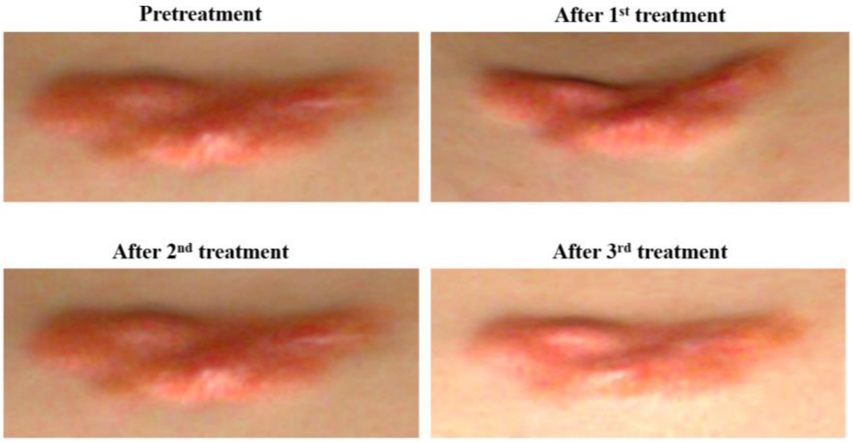

[Enter a number from 0 (very unwilling) to 10 (very willing)]*

____

15. If you are a scar patient, there is a treatment method that results in the effect shown in the picture after treatment, would you be willing to continue the treatment ?

VSS Score

(Note: Note: VSS score is a commonly used clinical rating for the severity of scars. The higher the total score, the more severe the scar)

VSS Score Items	Pretreatment	After 1st treatment	After 2nd treatment	After 3rd treatment
**Pigmentation (0- Close to skin color, 3- Darker color)**	0	0	0	0
**Vascularity (0- Close to skin color, 3- Bluish)**	1	1	1	1
**Height (0- Normal, 4- >4mm)**	2	2	2	2
**Pliability (0- Normal, 5- Contraction causing functional impairment)**	2	2	2	1
**Total Score**	5	5	5	4

[Enter a number from 0 (very unwilling) to 10 (very willing)]*

____

16. If you are a scar patient, there is a treatment method that results in the effect shown in the picture after treatment, would you be willing to continue the treatment ?

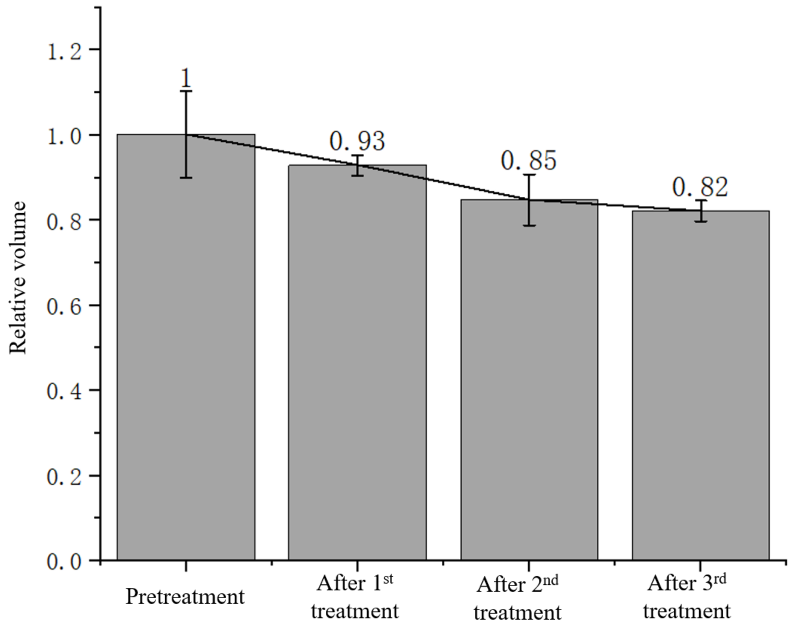

[Enter a number from 0 (very unwilling) to 10 (very willing)]*

____

17. If you are a scar patient, there is a treatment method that results in the effect shown in the picture after treatment, would you be willing to continue the treatment ?

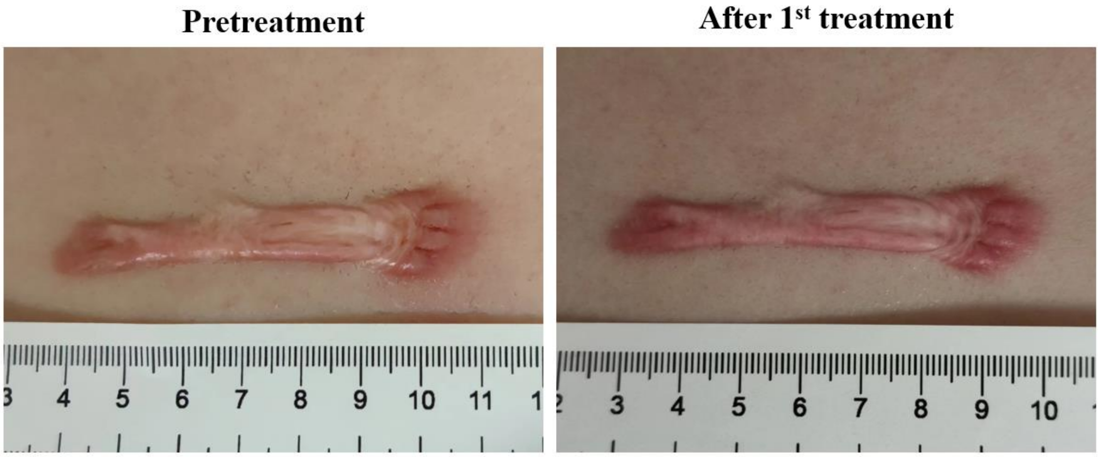

[Enter a number from 0 (very unwilling) to 10 (very willing)]*

____

18. If you are a scar patient, there is a treatment method that results in the effect shown in the picture after treatment, would you be willing to continue the treatment ?

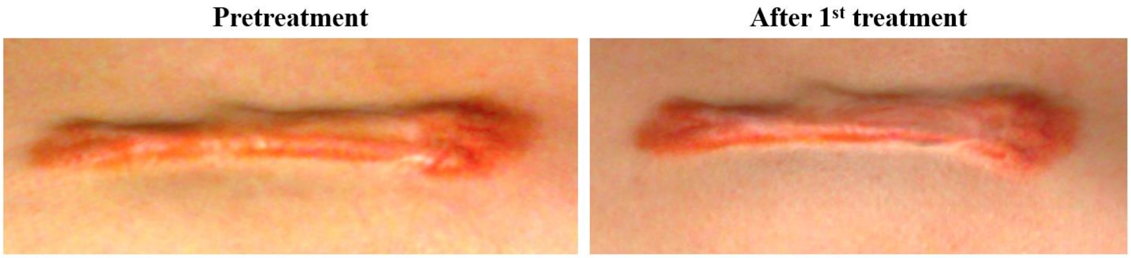

[Enter a number from 0 (very unwilling) to 10 (very willing)]*

____

19. If you are a scar patient, there is a treatment method that results in the effect shown in the picture after treatment, would you be willing to continue the treatment ?

VSS Score

(Note: Note: VSS score is a commonly used clinical rating for the severity of scars. The higher the total score, the more severe the scar)

VSS Score Items	Pretreatment	After 1st treatment
**Pigmentation (0- Close to skin color, 3- Darker color)**	1	1
**Vascularity (0- Close to skin color, 3- Bluish)**	1	1
**Height (0- Normal, 4- >4mm)**	3	3
**Pliability (0- Normal, 5- Contraction causing functional impairment)**	2	2
**Total Score**	7	7

[Enter a number from 0 (very unwilling) to 10 (very willing)]*

____

20. If you are a scar patient, there is a treatment method that results in the effect shown in the picture after treatment, would you be willing to continue the treatment ?

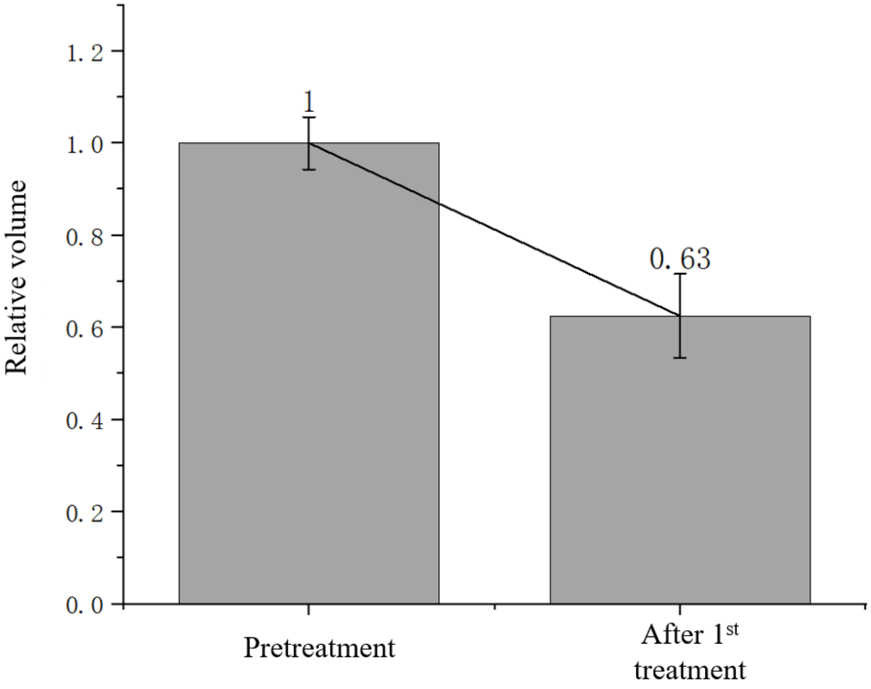

[Enter a number from 0 (very unwilling) to 10 (very willing)]*

____

21. If you are a scar patient, there is a treatment method that results in the effect shown in the picture after treatment, would you be willing to continue the treatment ?

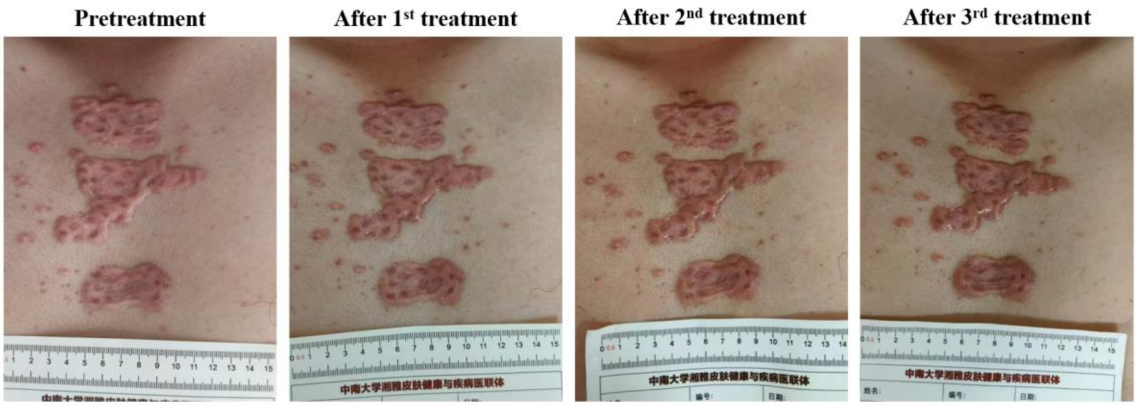

[Enter a number from 0 (very unwilling) to 10 (very willing)]*

____

22. If you are a scar patient, there is a treatment method that results in the effect shown in the picture after treatment, would you be willing to continue the treatment ?

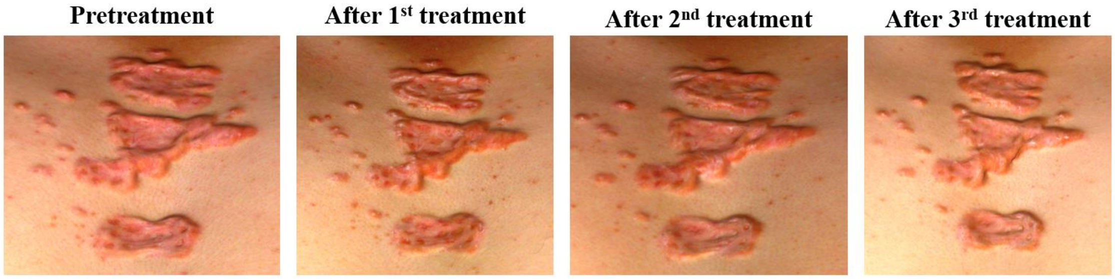

[Enter a number from 0 (very unwilling) to 10 (very willing)]*

____

23. If you are a scar patient, there is a treatment method that results in the effect shown in the picture after treatment, would you be willing to continue the treatment ?

VSS Score

(Note: Note: VSS score is a commonly used clinical rating for the severity of scars. The higher the total score, the more severe the scar)

VSS Score Items	Pretreatment	After 1st treatment	After 2nd treatment	After 3rd treatment
**Pigmentation (0- Close to skin color, 3- Darker color)**	2	2	2	2
**Vascularity (0- Close to skin color, 3- Bluish)**	2	2	2	2
**Height (0- Normal, 4- >4mm)**	3	3	3	3
**Pliability (0- Normal, 5- Contraction causing functional impairment)**	2	2	2	2
**Total Score**	9	9	9	9

[Enter a number from 0 (very unwilling) to 10 (very willing)]*

____

24. If you are a scar patient, there is a treatment method that results in the effect shown in the picture after treatment, would you be willing to continue the treatment ?

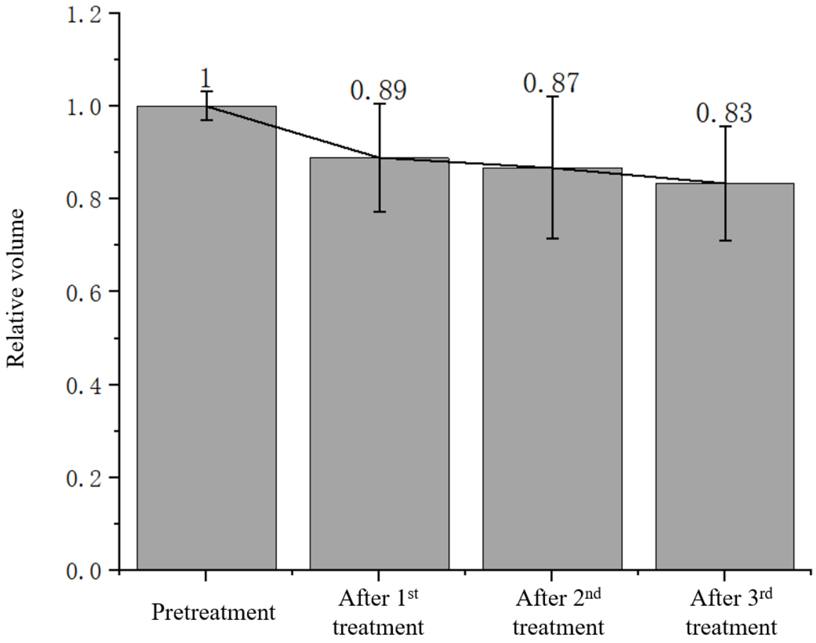

[Enter a number from 0 (very unwilling) to 10 (very willing)]*

____

25. If you are a scar patient, there is a treatment method that results in the effect shown in the picture after treatment, would you be willing to continue the treatment ?

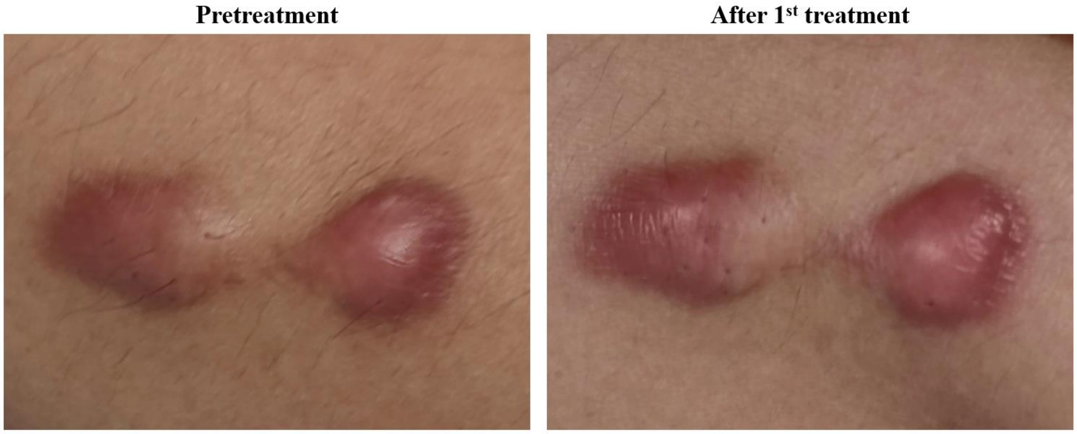

[Enter a number from 0 (very unwilling) to 10 (very willing)]*

____

26. If you are a scar patient, there is a treatment method that results in the effect shown in the picture after treatment, would you be willing to continue the treatment ?

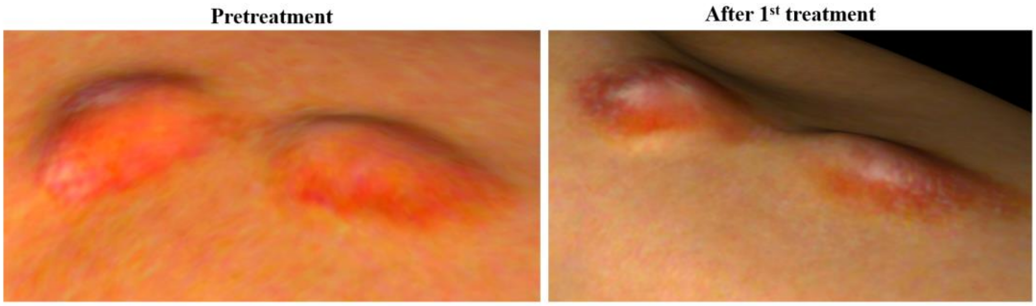

[Enter a number from 0 (very unwilling) to 10 (very willing)]*

____

27. If you are a scar patient, there is a treatment method that results in the effect shown in the picture after treatment, would you be willing to continue the treatment ?

VSS Score

(Note: Note: VSS score is a commonly used clinical rating for the severity of scars. The higher the total score, the more severe the scar)

VSS Score Items	Pretreatment	After 1st treatment
**Pigmentation (0- Close to skin color, 3- Darker color)**	1	1
**Vascularity (0- Close to skin color, 3- Bluish)**	3	3
**Height (0- Normal, 4- >4mm)**	4	4
**Pliability (0- Normal, 5- Contraction causing functional impairment)**	3	3
**Total Score**	11	11

[Enter a number from 0 (very unwilling) to 10 (very willing)]*

____

28. If you are a scar patient, there is a treatment method that results in the effect shown in the picture after treatment, would you be willing to continue the treatment ?

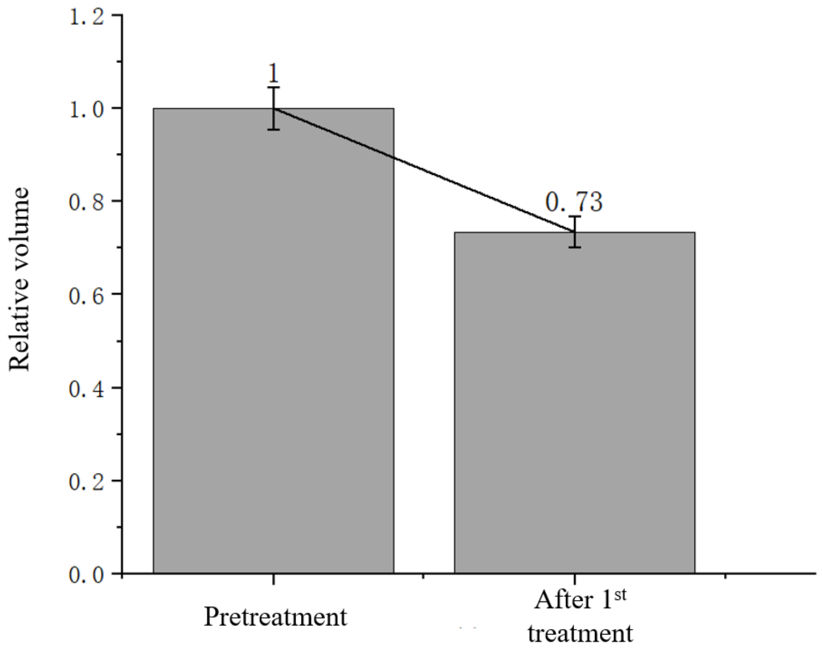

[Enter a number from 0 (very unwilling) to 10 (very willing)]*

____

29. If you are a scar patient, there is a treatment method that results in the effect shown in the picture after treatment, would you be willing to continue the treatment ?

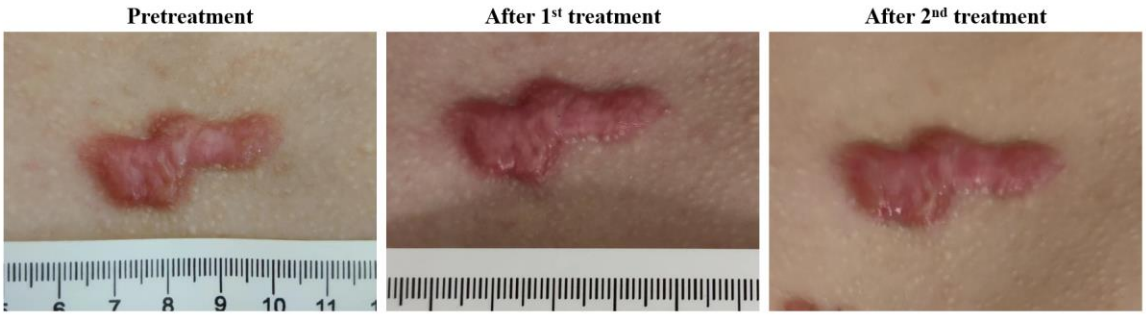

[Enter a number from 0 (very unwilling) to 10 (very willing)]*

____

30. If you are a scar patient, there is a treatment method that results in the effect shown in the picture after treatment, would you be willing to continue the treatment ?

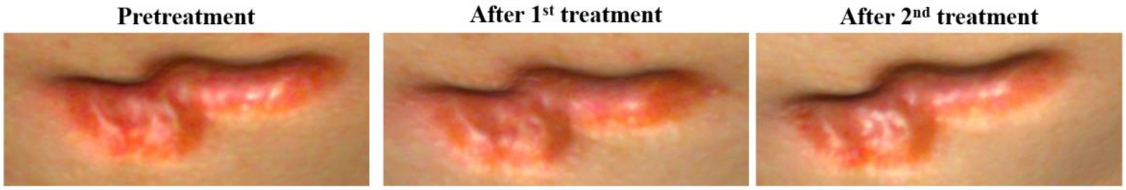

[Enter a number from 0 (very unwilling) to 10 (very willing)]*

____

31. If you are a scar patient, there is a treatment method that results in the effect shown in the picture after treatment, would you be willing to continue the treatment ?

VSS Score

(Note: Note: VSS score is a commonly used clinical rating for the severity of scars. The higher the total score, the more severe the scar)

VSS Score Items	Pretreatment	After 1st treatment	After 2nd treatment
**Pigmentation (0- Close to skin color, 3- Darker color)**	1	1	1
**Vascularity (0- Close to skin color, 3- Bluish)**	3	3	3
**Height (0- Normal, 4- >4mm)**	3	3	3
**Pliability (0- Normal, 5- Contraction causing functional impairment)**	3	3	3
**Total Score**	10	10	10

[Enter a number from 0 (very unwilling) to 10 (very willing)]*

____

32. If you are a scar patient, there is a treatment method that results in the effect shown in the picture after treatment, would you be willing to continue the treatment ?

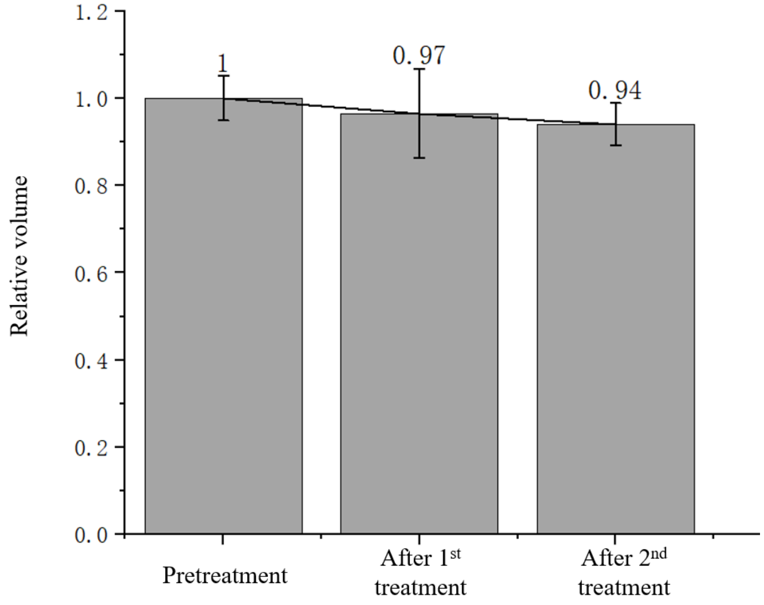

[Enter a number from 0 (very unwilling) to 10 (very willing)]*

____

33. If you are a scar patient, there is a treatment method that results in the effect shown in the picture after treatment, would you be willing to continue the treatment ?

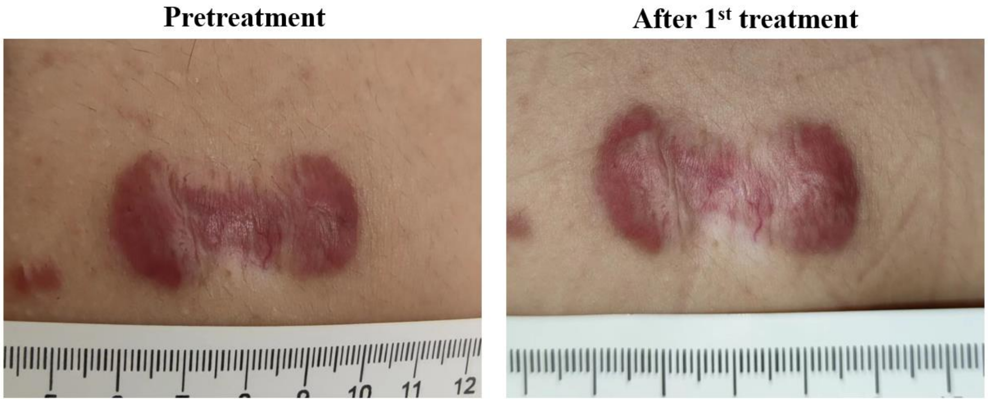

[Enter a number from 0 (very unwilling) to 10 (very willing)]*

____

34. If you are a scar patient, there is a treatment method that results in the effect shown in the picture after treatment, would you be willing to continue the treatment ?

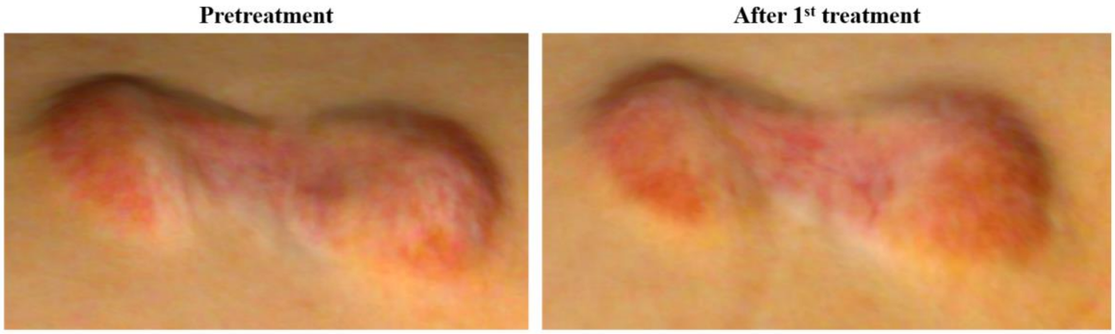

[Enter a number from 0 (very unwilling) to 10 (very willing)]*

____

35. If you are a scar patient, there is a treatment method that results in the effect shown in the picture after treatment, would you be willing to continue the treatment ?

VSS Score

(Note: Note: VSS score is a commonly used clinical rating for the severity of scars. The higher the total score, the more severe the scar)

VSS Score Items	Pretreatment	After 1st treatment
**Pigmentation (0- Close to skin color, 3- Darker color)**	1	1
**Vascularity (0- Close to skin color, 3- Bluish)**	3	3
**Height (0- Normal, 4- >4mm)**	1	1
**Pliability (0- Normal, 5- Contraction causing functional impairment)**	1	1
**Total Score**	6	6

[Enter a number from 0 (very unwilling) to 10 (very willing)]*

____

36. If you are a scar patient, there is a treatment method that results in the effect shown in the picture after treatment, would you be willing to continue the treatment ?

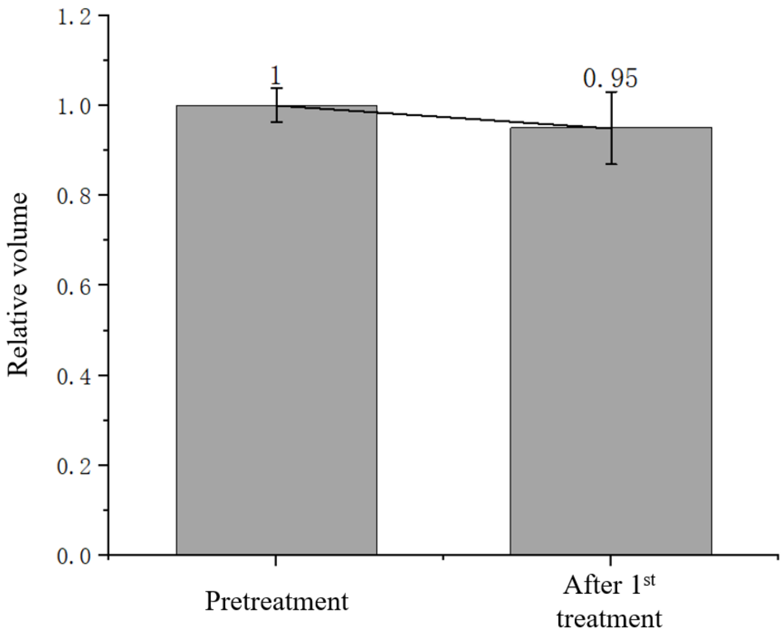

[Enter a number from 0 (very unwilling) to 10 (very willing)]*

____

37. If you are a scar patient, there is a treatment method that results in the effect shown in the picture after treatment, would you be willing to continue the treatment ?

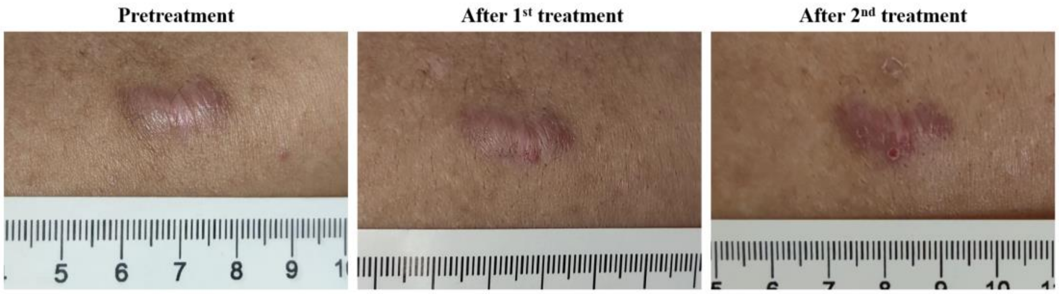

[Enter a number from 0 (very unwilling) to 10 (very willing)]*

____

38. If you are a scar patient, there is a treatment method that results in the effect shown in the picture after treatment, would you be willing to continue the treatment ?

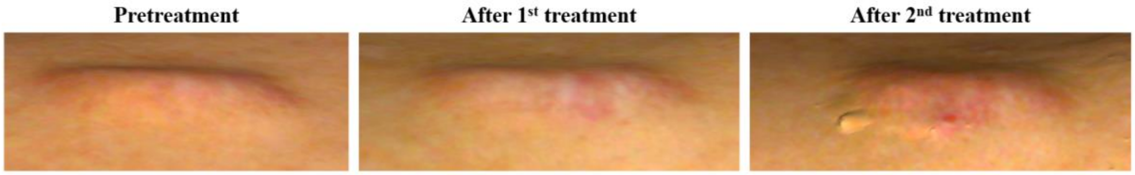

[Enter a number from 0 (very unwilling) to 10 (very willing)]*

____

39. If you are a scar patient, there is a treatment method that results in the effect shown in the picture after treatment, would you be willing to continue the treatment ?

VSS Score

(Note: Note: VSS score is a commonly used clinical rating for the severity of scars. The highe[illegible] the total score, the more severe the scar)

VSS Score Items	Pretreatment	After 1st treatment	After 2nd treatment
**Pigmentation (0- Close to skin color, 3- Darker color)**	1	1	1
**Vascularity (0- Close to skin color, 3- Bluish)**	3	3	3
**Height (0- Normal, 4- >4mm)**	2	2	2
**Pliability (0- Normal, 5- Contraction causing functional impairment)**	3	3	3
**Total Score**	9	9	9

[Enter a number from 0 (very unwilling) to 10 (very willing)]*

____

40. If you are a scar patient, there is a treatment method that results in the effect shown in the picture after treatment, would you be willing to continue the treatment ?

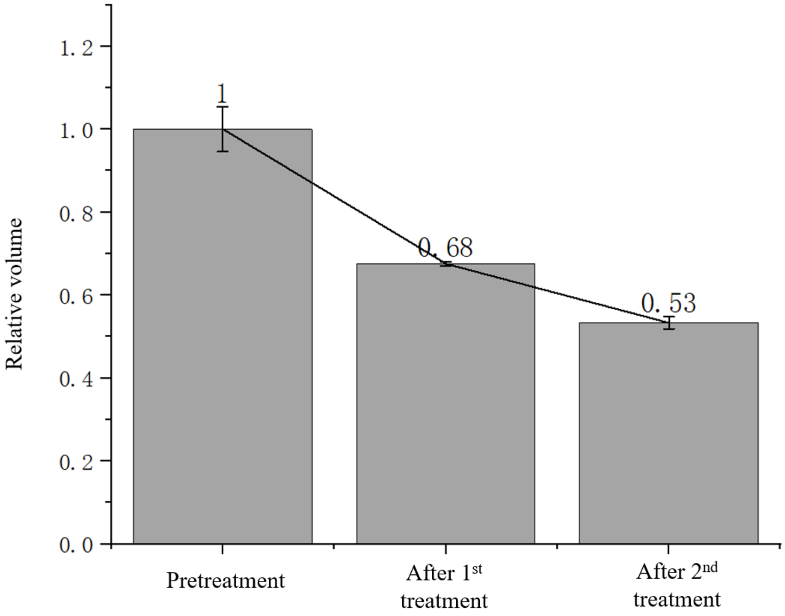

[Enter a number from 0 (very unwilling) to 10 (very willing)]*

____

41. If you are a scar patient, there is a treatment method that results in the effect shown in the picture after treatment, would you be willing to continue the treatment ?

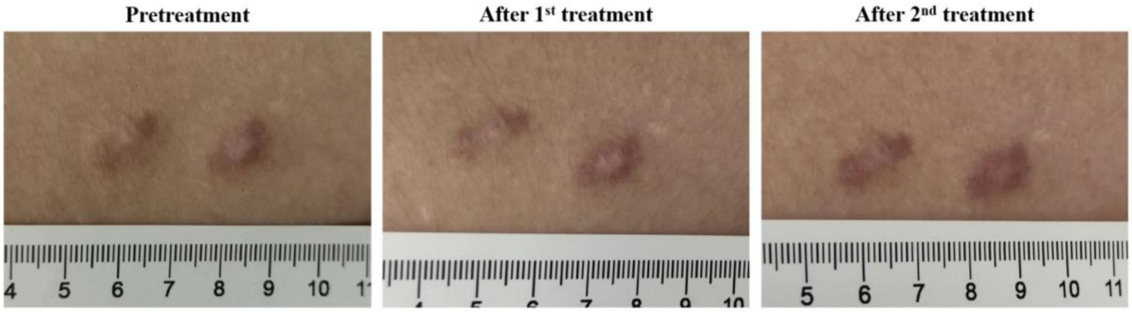

[Enter a number from 0 (very unwilling) to 10 (very willing)]*

42. If you are a scar patient, there is a treatment method that results in the effect shown in the picture after treatment, would you be willing to continue the treatment ?

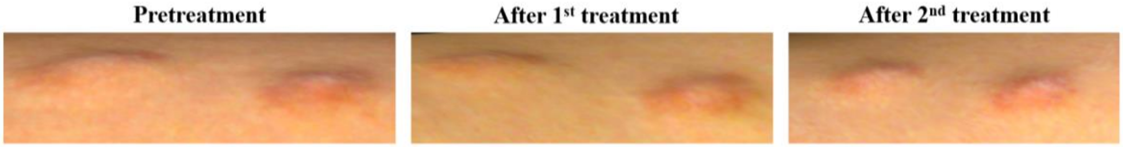

[Enter a number from 0 (very unwilling) to 10 (very willing)]*

43. If you are a scar patient, there is a treatment method that results in the effect shown in the picture after treatment, would you be willing to continue the treatment ?

VSS Score

(Note: Note: VSS score is a commonly used clinical rating for the severity of scars. The higher the total score, the more severe the scar)

VSS Score Items	Pretreatment	After 1st treatment	After 2nd treatment
**Pigmentation (0- Close to skin color, 3- Darker color)**	1	1	1
**Vascularity (0- Close to skin color, 3- Bluish)**	3	3	3
**Height (0- Normal, 4- >4mm)**	1	1	1
**Pliability (0- Normal, 5- Contraction causing functional impairment)**	3	3	3
**Total Score**	8	8	8

[Enter a number from 0 (very unwilling) to 10 (very willing)]*

____

44. If you are a scar patient, there is a treatment method that results in the effect shown in the picture after treatment, would you be willing to continue the treatment ?

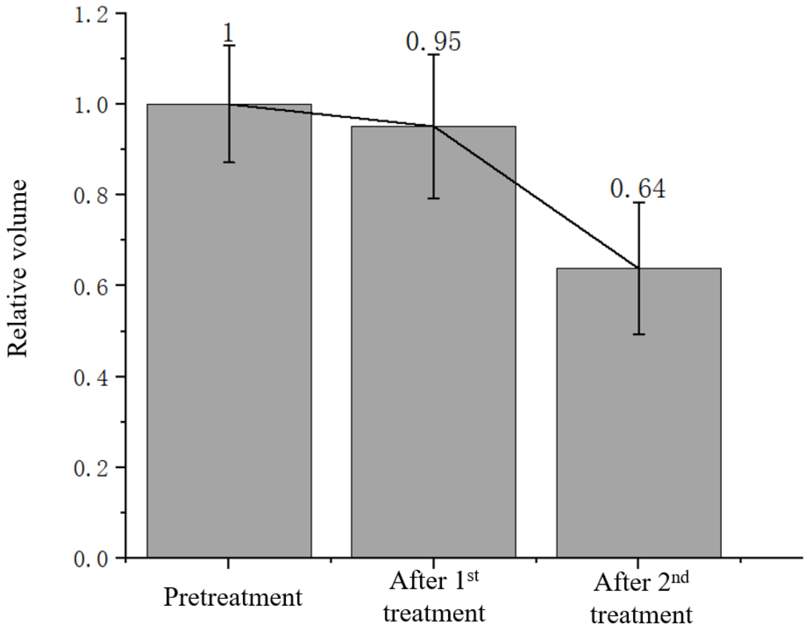

[Enter a number from 0 (very unwilling) to 10 (very willing)]*

____
